# The Reparative Mechanisms Underlying Preclinical Neural Stem/Progenitor Cell Therapy for Ischemic Stroke: A Systematic Review

**DOI:** 10.1155/nri/9430707

**Published:** 2026-08-29

**Authors:** Joel Karikari Nyarkoh, Ernest Amponsah Asiamah, Samuel Badu Nyarko, Joseph Boachie, Christian Dzuvor, Gabriel Kotam Pezahso, Moses Tetteh Larnyoh, Obed Akorli Mawunyo, Bernard Nyarko

**Affiliations:** ^1^ Department of Medical Laboratory Sciences, School of Allied Health Sciences, College of Health and Allied Sciences, University of Cape Coast, Cape Coast, Ghana, ucc.edu.gh; ^2^ Department of Biomedical Sciences, School of Allied Health Sciences, College of Health and Allied Sciences, University of Cape Coast, Cape Coast, Ghana, ucc.edu.gh; ^3^ Department of Medical Biochemistry, School of Medical Sciences, College of Health and Allied Sciences, University of Cape Coast, Cape Coast, Ghana, ucc.edu.gh; ^4^ Department of Chemical Engineering, College of Engineering, Kwame Nkrumah University of Science and Technology, Kumasi, Ghana, knust.edu.gh; ^5^ Department of Imaging Technology and Sonography, School of Allied Health Sciences, College of Health and Allied Sciences, University of Cape Coast, Cape Coast, Ghana, ucc.edu.gh

**Keywords:** bystander effect, ischemic stroke, neural stem/progenitor cells, neurogenesis, neurorestoration

## Abstract

**Background:**

Ischemic stroke is a major cause of death and disability worldwide. Current treatments, such as thrombolysis and mechanical thrombectomy, restore perfusion but do not promote tissue repair, highlighting the need for regenerative therapies. Neural stem/progenitor cells (NSPCs) have been extensively explored in preclinical models; however, the mechanisms underlying their therapeutic effects remain incompletely defined.

**Methods:**

This study followed the PRISMA guidelines to address the cellular and molecular mechanisms that underlie the neurorestorative effects of transplanted NSPCs in animal models of ischemic stroke. Preclinical studies published between 2013 and 2023 were identified through PubMed, Scopus, and Google Scholar. Eligible studies compared NSPC monotherapy with vehicle‐treated controls, and data on transplantation protocols and functional, histological, and biochemical outcomes were extracted for narrative synthesis. Of the 8697 records identified, 25 met the inclusion criteria, with moderate methodological quality (median CAMARADES score: 6/10; range: 3–8).

**Results:**

NSPC transplantation significantly improved motor, sensory, and cognitive function while reducing infarct volume, neuronal apoptosis, neuroinflammation, and blood–brain barrier disruption, alongside increased neurotrophic and angiogenic factors (BDNF, GDNF, VEGF, and Ang‐1) and modulation of inflammatory mediators. Functional recovery was more consistently associated with paracrine and immunomodulatory mechanisms than with direct neuronal replacement, which occurred less frequently and was often accompanied by astrocytic or oligodendroglial differentiation; astrocytic responses were conflicting, suggesting context‐dependent effects. These preclinical mechanisms have not been consistently replicated in early‐phase human trials, pointing to immunogenicity and delivery route as key translational barriers.

**Conclusion:**

The reparative benefits of NSPC therapy in ischemic stroke are largely attributable to paracrine and immunomodulatory mechanisms, with neuronal replacement playing a minor role. Future studies should focus on strategies to potentiate paracrine activity, improve graft survival, and adopt translationally relevant, comorbidity‐inclusive models to advance clinical translation.

## 1. Introduction

Stroke is a neurological disease known for its unwarranted morbidity and mortality. It is the second leading cause of death and a pivotal contributor to disability worldwide [1]. These disabilities include cognitive impairment, somatosensory dysfunction, and paralysis [2]. Stroke is categorized into ischemic stroke and hemorrhagic stroke, of which ischemic stroke accounts for approximately 80%, highlighting the contribution of ischemic stroke to stroke‐induced impairments [3]. Ischemic stroke results from the occlusion of the cerebral arteries; the reduction and/or lack of perfusion causes infarctions in the subserved brain region(s). According to the Stroke Recovery and Rehabilitation Roundtable Task Force (SRRT, 2017), with reference to the onset of ischemic stroke, the progression of ischemic stroke is categorized into four phases, namely, hyperacute (0–24 h), acute (1–7 days), subacute (1 week–6 months) and chronic (older than 6 months) [4]. The hyperacute phase is characterized by neuronal death and the onset of inflammation. Inflammation continues in the acute phase and co‐occurs with glial scar formation. The acute phase culminates with the onset of endogenous plasticity and spontaneous improvement in neurobehavioral function, and both processes persist in the subacute and chronic phases of ischemic stroke [4]. All these activities highlight the co‐occurrence of both deteriorative and ameliorative processes in the pathophysiology of ischemic stroke. Thus, an intervention that can augment the recovery process and/or mitigate the deteriorative processes is a potential treatment option.

To date, the putative treatment options for ischemic stroke are thrombolysis and mechanical thrombectomy. Traditionally, recombinant tissue plasminogen activator (rtPA) has served as the primary thrombolytic agent for endovascular recanalization [5]. However, tenecteplase (TNK), a bioengineered alteplase derivative with enhanced fibrin selectivity and prolonged plasma half‐life, has gained endorsement in recent stroke management guidelines and is increasingly recognized as the drug of choice [6]. Despite these advances, thrombolysis has several shortcomings, including a narrow therapeutic time window (∼4.5 h), benefiting only 3% of ischemic stroke patients [7]. This treatment option can also cause ischemia–reperfusion injury, which has the potential to damage the blood–brain barrier (BBB), resulting in hemorrhage [8]. Mechanical thrombectomy, on the other hand, is a surgical procedure used for endovascular recanalization. Mechanical thrombectomy has a relatively longer time window of 6–24 h [9], compared to rtPA or TNK administration (thrombolysis). Both strategies can successfully achieve recanalization but are also challenged by ischemia–reperfusion injury. Moreover, neither thrombolytic agents (rtPA or TNK) nor mechanical thrombectomy facilitates tissue regeneration within the infarcted region, underscoring the necessity for reparative interventions such as stem cell–based therapies [6, 10].

Stem cell therapy, particularly using neural stem/progenitor cells (NSPCs), has emerged as a promising therapeutic option for managing ischemic stroke [11]. Neural stem cells (NSCs) and their immediate non‐stem–cell progeny, neural progenitor cells (NPCs), are multipotent cells; they proliferate limitlessly and differentiate into neurons and neuroglia [12]. Available studies have revealed the presence of NSPCs in brain regions affected by ischemic stroke, indicating their involvement in the disease process [13, 14]. During the disease process, stroke‐induced endogenous neurogenesis elicits vigorous proliferation and migration of NSPCs from adult neurogenic niches (SVZ and SGZ) toward the ischemic side, contributing to the recovery process. This observation, at least, has promoted the transplantation of NSPCs to augment the recovery process.

Initially, studies posited neuronal replacement as the primary mechanism of posttransplantation recovery [15, 16]. However, increasing evidence suggests that paracrine factors may play a more prominent role [17–19]. Zhang et al. (2023) found that several factors could influence the outcomes of NSPC transplantation, including the type of donor cells, cell dose, timing of administration after stroke, delivery route, and anesthesia. Similarly, Huang et al. (2018) reported that endogenous apoptosis inhibition and axonal regeneration played the most critical roles in ischemic stroke recovery [20]. Thus far, systematic reviews on the therapeutic potential of NSPCs in ischemic stroke have been published [12, 21], but each carries limitations that constrain their mechanistic conclusions. Huang et al. (2018) [20], for example, restricted their meta‐analysis to intraparenchymally delivered NSPCs and to neurobehavioral and infarct‐volume endpoints alone, excluding systemic delivery routes and biochemical/paracrine outcomes from quantitative synthesis; more broadly, existing reviews have generally described individual reparative pathways in isolation, without a formal, checklist‐based quality appraisal of the underlying preclinical evidence and without explicitly excluding studies confounded by cointerventions such as biomaterial scaffolds, genetic modification, or cotransplantation with other cell types. Consequently, the relative contribution of cell replacement versus paracrine bystander mechanisms to functional recovery has not been systematically synthesized within a single, quality‐appraised framework restricted to NSPC monotherapy. Unraveling the NSPC‐induced reparative mechanism is essential, as it provides foundational knowledge to enhance the ameliorative potential of NSPCs and to guide the rational, mechanism‐based development of future therapeutic strategies.

This systematic review, therefore, seeks to elucidate the reparative mechanisms underlying NSPC‐mediated recovery in preclinical models of ischemic stroke by synthesizing evidence from behavioral, histological, and biochemical outcomes. Specifically, the review addresses the research question: “What cellular and molecular mechanisms underlie the neurorestorative effects of transplanted NSPCs in animal models of ischemic stroke?” Its central contribution is methodological rather than discovery‐based: by integrating functional, structural, and biochemical evidence within a single, quality‐appraised framework restricted exclusively to unmodified NSPC monotherapy, and by explicitly cataloging points of disagreement across studies rather than presenting a uniform narrative, this review aims to weigh the relative contribution of candidate reparative mechanisms in a way that no prior review of this literature has attempted.

## 2. Method

### 2.1. Study Design and Registration

This study was conducted in line with the Preferred Reporting Items for Systematic Reviews and Meta‐Analyses (PRISMA) guidelines [22]. The protocol was registered in the International Prospective Register of Systematic Reviews (PROSPERO; registration number CRD420261442462).

### 2.2. Eligibility Criteria

To avert selection bias, inclusion criteria were prespecified for experimental studies in which the study population was categorized into treatment and control groups. Both groups underwent the same stroke induction procedure followed by transplantation of NSPCs in the treatment group, while the control group received a nonneuroprotective vehicle. Studies were excluded if the stroke model was hemorrhagic, if they were published in a language other than English, if NSPC therapy involved cells with gene modification or utilized a bio‐scaffold as a delivery vehicle, or if NSPCs were cotransplanted with other stem cell types, stem cell derivatives, or neuroprotective drugs. These rigorous inclusion criteria were deliberately implemented to reduce confounding variables and isolate the intrinsic reparative capacity of NSPCs as a monotherapy. Studies involving combinatorial approaches, such as biomaterial scaffolds, cotransplantation strategies, or genetic modifications, were excluded to preserve methodological consistency and reinforce the internal validity of mechanistic interpretations. Studies were excluded if they were review articles, editorials, letters, conference abstracts, non‐English publications, or studies without extractable data.

### 2.3. Search Strategy

A comprehensive literature search was conducted in PubMed, Scopus, and Google Scholar from January 1, 2013, to December 31, 2023. The search strategy combined free‐text terms related to ischemic stroke and NSPCs, including the following: “stroke,” “cerebral ischemia,” “cerebrovascular accident,” “middle cerebral artery occlusion,” “cerebral photothrombosis,” “stem cells,” “induced pluripotent stem cell‐derived neural stem cells,” “neural stem cells,” and “neural progenitor cells.” For Google Scholar, the search strategy was as follows: (stroke) OR (cerebral ischemia) OR (cerebrovascular accident) OR (middle cerebral artery occlusion) OR (cerebral photothrombosis) AND (stem cells) OR (induced pluripotent stem cells‐derived neural stem cells) OR (neural stem cells) OR (neural progenitor cells). The search strategy was adapted for each database to ensure consistency in the retrieval of relevant studies. No restrictions were applied with respect to animal species or country of study origin. Only full‐text studies published in English were included.

### 2.4. Study Selection

**FIGURE 1:**
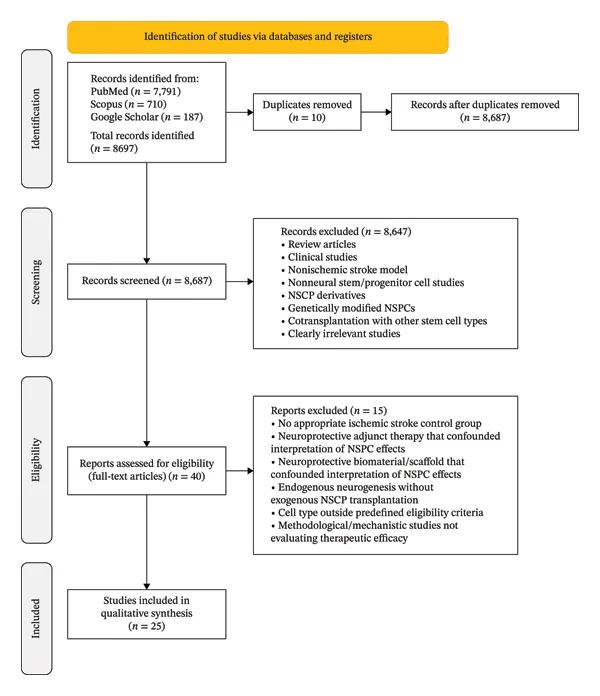
PRISMA flow diagram of data search procedure.

All records identified through database searching were imported into Rayyan QCRI for screening, and duplicates were removed before analysis. The search retrieved a total of 8697 records, including PubMed (*n* = 7791), Scopus (*n* = 710), and Google Scholar (*n* = 187). After deduplication, 8687 records remained for title and abstract screening. Titles and abstracts were independently screened by two reviewers to identify potentially eligible studies. Full‐text articles of 40 records were then assessed for eligibility against the predefined inclusion criteria. Discrepancies at any stage of screening were resolved through discussion and consensus. Additional studies were identified through manual screening of reference lists of included articles and relevant reviews. Following full‐text assessment, 25 studies [23–47] met all eligibility criteria and were included in the final systematic review. The study selection process is presented in the PRISMA flow diagram (Figure 1).

### 2.5. Data Extraction

Data were extracted using a standardized data extraction form. Extracted variables included author name, publication year, source of cell lines, cell immunogenicity, the vehicle used, species receiving NSPCs, sex, cell dose, route of administration, time of cell administration, duration of treatment, technique for stroke induction, anesthesia, immunosuppressant, structural outcome measured (including infarct volume, immunohistochemistry, and neuropathological changes), functional outcome measured (namely, motor functions, sensory function, and cognitive functions), and biochemical outcomes (namely, neurotrophic factors, growth factors, cytokines, chemokines, and cell adhesion molecules). Where studies reported multiple outcome subgroups, data were extracted separately where possible. All extracted data were cross‐checked for consistency before synthesis.

### 2.6. Quality Assessment

Each included study was assessed for quality using the modified CAMARADES checklist (maximum score = 10), which evaluates key elements of internal and external validity in preclinical studies [48]. The median quality score of the included studies was 6 (range 3–8), indicating moderate methodological rigor. While the number of studies included was smaller than in broader narrative reviews, our approach prioritized methodological integrity and comparability over volume, thereby enhancing the reliability of synthesized findings (Table 1).

**TABLE 1:** Proportion of the included studies (*N* = 25) meeting each quality score criterion.

Quality score criterion	Studies meeting criterion%
Published in a peer‐reviewed journal	100
Statement confirming compliance with animal welfare requirements	96
Control of temperature	92
Avoided neuroprotective anesthetics	88
Conflict of interest statement	72
Random treatment assignment	64
Blinded outcomes	64
Sample size calculation	8
Animals with comorbidities	4
Allocation concealment	0

### 2.7. Data Synthesis

Data extracted from eligible studies were synthesized narratively. Study characteristics, including publication year, source and species of NSPCs, species receiving transplantation, sex, cell immunogenicity, vehicle used, dose range, route and timing of administration, mechanism of stroke induction, and anesthesia, were summarized in descriptive tables using frequencies and percentages. Study quality was assessed using a modified CAMARADES checklist, and the proportion of studies meeting each quality criterion was summarized descriptively, alongside the median and range of overall quality scores.

Outcomes were synthesized narratively across three domains: functional/neurobehavioral outcomes (motor, sensory, and cognitive recovery), structural/histological outcomes (infarct volume, apoptosis, angiogenesis, BBB integrity, microgliosis, and astrogliosis), and biochemical/paracrine outcomes (neurotrophic, growth, inflammatory, and cell‐adhesion factors). For each domain, findings were tabulated by study, reporting the specific outcome measure or marker used, the phase of stroke at treatment (hyperacute, acute, or subacute), and the direction and statistical significance of the effect in the NSPC‐treated group relative to vehicle‐treated controls. Where studies reported conflicting findings for the same outcome, these discrepancies were highlighted and considered in relation to differences in stroke phase, cell source, or outcome marker across studies. A quantitative meta‐analysis was not performed owing to substantial clinical and methodological heterogeneity across included studies, including variation in animal species and strains, mechanisms of stroke induction, NSPC source and dose, route and timing of administration, and the diversity of outcome measures and assessment tools used for comparable constructs. This heterogeneity precluded meaningful statistical pooling of effect sizes.

## 3. Results

### 3.1. Study Characteristics

Among the 25 included studies, 14 (56%) were published between 2013 and 2017 [23–32, 39, 42, 45], and 11 (44%) were published between 2018 and 2023 [34–38, 40, 41, 43, 44, 46, 47]. Humans, mice, and rats were the primary sources of NSPCs according to 13 (52%), 8 (32%), and 4 (16%) studies, respectively. NSCs were used in 15 (60%) of the included studies, whereas the remaining 10 (40%) studies used NPCs. Mice and rats were the predominant animal species used for these preclinical studies (11 and 12 studies, respectively). Almost all studies used male animals (24, 96%). Most studies used middle cerebral artery occlusion (MCAo) to induce ischemic stroke (22; 88%). Most of the treatments were administered at the acute phase (1–7 days) of ischemic stroke, and the intracerebral route was the most frequently used 12 (48%). The predominant dose range was (1 × 10^5^–6 × 10^5^) cells. Concerning transplant immunogenicity, 14 (56%) studies reported xenogeneic transplantation, and 11 (44%) studies reported allogeneic transplantation (Table 2).

**TABLE 2:** Characteristics of the 25 studies included in the review.

Characteristics	Summary data
*Study characteristics*	
Number of publications	25
Year of publication	
2013–2017	14 (56%)
2018–2023	11 (44%)

*Animal characteristics*	
Transplanted cells	
NSCs	15 (60%)
NPCs	10 (40%)
Source of NSPCs	
Human	13 (52%)
Mouse	8 (32%)
Rat	4 (16%)
Species receiving the NSPCs	
Rat	12 (48%)
Mouse	11 (44%)
Pig	2 (8%)
Sex	
Males only	24 (96%)
Females only	0 (0%)
Both	1 (4%)

*Stroke type characteristics*	
Mechanism of stroke induction	
MCAo	22 (88%)
ET‐1	2 (8%)
Photothrombosis	1 (4%)

*Treatment characteristics*	
Cell immunogenicity	
Xenogeneic	14 (56%)
Allogeneic	11 (44%)
Autologous	0
Vehicle used	
PBS	13 (52%)
DMEM	2 (8%)
aCSF	2 (8%)
Saline	1 (4%)
not specified	1 (4%)
Dose range (*n* = 29, more than 1 experiment in a study)	
< 100,000 cells	4 (13.8%)
100,000–600,000 cells	15 (51.7%)
1,000,000–1,500,000 cells	9 (31.0%)
10,000,000 cells	1 (3.5%)
Route of administration	
Intracerebral	12 (48%)
Intravenous	5 (20%)
Intrahippocampal	4 (16%)
Intraventricular	2 (8%)
Epidural	1 (4%)
Intra‐arterial	1 (4%)
Time of cell administration poststroke. (*n* = 33, more than one experiment in a study)	
≤ 12 hours	6 (18.2%)
24 hours	8 (24.2%)
> 24 hours–6 days	14 (42.4%)
≥ 1 week	5 (15.2%)
Anesthesia	
Isoflurane	14 (56%)
Chloral hydrate	3 (12%)
Ketamine/xylazine	4 (16%)
Not specified	4 (16%)

*Note:* Data are represented as studies or experiments *n* (%) with that specific characteristic.

Abbreviations: aCSF, artificial cerebrospinal fluid; DMEM, Dulbecco’s modified Eagle’s media; ET‐1, endothelin‐1; MCAo, middle cerebral artery occlusion; NPCs, neural progenitor cells; NSCs, neural stem cells; NSPCs, neural stem/progenitor cells; PBS, phosphate‐buffered saline.

### 3.2. Evidence of Improved Neurobehavioral Functions

Neurobehavioral tests included in the studies were the rotarod test, cylinder test, adhesive removal test, foot fault test, and neurological severity score for lissencephalic animals (rats and mice) and the poststroke assessment scale for gyrencephalic animals (pigs). Most studies showed significantly enhanced neurobehavioral functional recovery when NSPCs were administered at the acute phase of the ischemic stroke, while only one study reported a similar outcome when treatment was administered at the subacute phase of stroke (28 days after onset) [26]. Compared with vehicle transplantation, NSPC transplantation led to a significant improvement in motor coordination as determined by the rotarod test (*p* < 0.001) [33, 47], somatosensory function as determined by the adhesive removal test (*p* < 0.001) [27, 47], limb use asymmetry as determined by the cylinder test (*p* < 0.001) [47] and mitigation of neurological deficits as determined by NSS (*p* < 0.001) [36] (Table 3).

**TABLE 3:** Evidence of improved neurobehavioral functions.

Study (year)	Behavioral test	Outcome measured	Time point	*p* value
Doeppner et al. 2014 [26]	Rotarod test	Time on the rod (sec)	84dps/56dpt (sA)	*P* < 0.05^#^

Tajiri et al. 2014 [31]	mNSS	Mean score/3	56dps/49dpt (A)	*P* < 0.05^∗^

Lee et al. 2017 [28]	Cylinder test	Ipsilesional forelimb use bias	24dps/14dpt (A)	*P* < 0.05^∗^

Doeppner et al. 2014B [25]	Rotarod test	Time on the rod (sec)	84dps/83.75dpt (hA)	*P* < 0.05^#^
	Adhesive removal	Contralateral removal time (sec)		*P* < 0.05^∗^

Lau et al. 2018 [44]	Poststroke assessment scale	Score/30	17dps/12dpt (A)	*P* < 0.05^∗^

Ryu et al. 2016 [30]	mNSS	Score/20	14dps/13dpt (A)	*P* < 0.01^∗^

Huang et al. 2014 [27]	Rotarod test	Time on the rod (sec)	24dps/23dpt (A)	*P* < 0.01^#^
	Adhesive removal	Removal time (sec)	4dps/3dpt (A)	*P* < 0.001^∗^

Tang et al. 2014 [23]	Rotarod test mNSS	Time on the rod (sec)	14dps/13dpt (A)	*P* < 0.05^#^
		Score/14		*P* < 0.05^∗^

Ma et al. 2015 [29]	Rotarod test	Latency (sec)	28dps/26dpt (A)	*P* < 0.01^#^

Yamashita et al. 2017 [32]	NSS (Bederson’s)	Score/4	8dps/7dpt (A)	*P* < 0.05^∗^

Ziaee et al. 2017 [42]	NSS	Score/5	31dps/28dpt (A)	*P* < 0.05^∗^

Yao et al. 2015 [39]	mNSS	Score/15	14dps/12dpt (A)	*P* < 0.05^∗^
	Adhesive removal	Removal time (sec)		

Islam et al. 2021 [35]	Foot fault	% slippage ratio	20dps/16dpt (A)	*P* < 0.05^∗^

Eckert et al. 2015 [33]	Rotarod test	Time on the rod (sec)	24dps/23dpt (A)	*P* < 0.001^#^
	Adhesive removal	Removal time (sec)	4dps/3dpt (A)	*P* < 0.01^∗^

Kondori et al. 2020 [36]	NSS	Score/5	8dps/7dpt (A)	*P* < 0.001^∗^

Yin et al. 2023 [40],	NSS (Clarke)	Score/‐	3dps/3dpt (hA)	*P* < 0.01^∗^

Vonderwalde et al. 2020 [38]	Foot fault test	% fault difference	32dps/28dpt (A)	*P* < 0.05^∗^

Hermanto et al. 2018 [34]	Cylinder test	Limb use bias	32dps/30dpt (hA)	*P* = 0.002^∗^

Palma et al. 2020 [46]	Cylinder test	Limb use bias	6mps–2dps (A)	*P* < 0.05^∗^

Tahta et al. 2018 [47]	Adhesive removal	Removal time (sec)	28dps/27dpt (A)	*P* < 0.001^∗^
	Cylinder test	Limb use bias		*P* < 0.001^#^
	Rotarod test	Time on the rod (sec)		

Lu et al. 2017 [45]	NSS	Score/14	14dps/10dpt (A)	*P* < 0.01^∗^

*Note:* The phase of stroke based on “the duration of stroke prior to treatment” (N[dps]–N[dpt]): hyperacute (hA) = 0–24 h, acute (A) = 1–7 days, and subacute (sA) = 1 week–6 months. sec: seconds.

Abbreviations: dps = day(s) poststroke, dpt = day(s) posttreatment, (m) NSS = (modified) neurological severity score.

^#^A significant increase in the measured outcome led to improvement in the behavioral test in the treatment group compared to the vehicle group.

^∗^A significant decrease in the measured outcome led to improvement in the behavioral test in the treatment group compared to the vehicle group.

### 3.3. Evidence of Improved Histological Outcomes

NSPC transplantation during both the acute and subacute phases of ischemic stroke [26] significantly improved histological outcomes. Compared to the vehicle, NSPC transplantation increased the population of mature neurons (NeuN + cells) [26], immature neurons (Dcx + cells) [26, 30], oligodendrocytes (MBP+/NG2+cells) [28], and astroglial cells (GFAP + cells) [47]. All cells demonstrated bromodeoxyuridine (BrdU) positivity. Also, NSPC transplantation led to a decrease in TUNEL + cells, indicating a decrease in neuronal death/apoptosis. Additionally, compared with vehicle treatment, NSPC treatment reduced infarct volume (MR‐T2W images, *p* < 0.0001) [47], enhanced BBB integrity or stability (> ZO‐1 and < MMP‐9, *p* < 0.001) [27, 33], decreased microglia activity (< Iba + cells and < MIP‐1*α*, *p* < 0.001) [33] and reduced leukocyte infiltration (< NK + cells, *p* < 0.001) [40] and improved angiogenesis (> CD3+ cells, *p* < 0.001) [45]. Most studies, especially those involving acute‐phase treatment, that assessed the presence of astrocytes (GFAP + cells) found a reduced number of astrocytes, except for one that found an increased number of astrocytes (> GFAP + cells, *p* < 0.0001) [47] (Table 4).

**TABLE 4:** Evidence of improved histological outcomes.

Study (year)	Animal used	Structural outcome measured	Marker/stain	Time point	*p* value
Doeppner et al. 2014 [26]	C57BL6/J mice	Infarct volume	CV/TTC	2dps/1dpt (A)	*P* < 0.05^∗^
		Apoptosis	TUNEL+		
		BBB stability	EBE		
		Microglia activity	IB4+		
		Axonal density	BDA‐DAB	84dps/56dpt (sA)	*P* < 0.05^#^
		Neurogenesis	BrdU+/Dcx/NeuN+		
		Angiogenesis	BrdU+/CD31+		

Lee et al. 2017 [28]	Long–Evans rats	Infract volume	H&E	28dpt/21dpt (A)	*P* < 0.05^∗^
		Microglia activity	ED+		
		Apoptosis	TUNEL+		*P* < 0.01^∗^
		Astrogliosis	GFAP+		
		Neurogenesis	BrdU+/MBP+/NG2+		*P* < 0.05^#^
		Angiogenesis	RECA+		

Doeppner et al. 2014B [25]	C57BL6 mice	Infarct volume	TTC	2dps/1.75dpt(hA)	*P* < 0.05^∗^
		Apoptosis	TUNEL+		
		Microglia activity	IB4+		

Ryu et al. 2016 [30]	Sprague–Dawley rats	Infarct volume	CV	14dps/13dpt (A)	*P* < 0.05^∗^
		Angiogenesis	BrdU+/vWF+		*P* < 0.05^#^
		Neurogenesis	BrdU+/Dcx+/GFAP+		

Huang et al. 2014 [27]	C57BL/6J mice	Infarct volume	CV/TTC staining	2dps/1dpt (A)	*P* < 0.05^∗^
		BBB stability	MMP‐9, ZO‐1,		*P* < 0.01^∗^, *P* < 0.001^#^
		Microglia activity	IgG staining		*P* < 0.05^∗^
			MCP‐1, MIP‐1*α*		*P* < 0.001^∗^, *P* < 0.01^∗^

Baker et al. 2017 [24]	Landrace pigs	Neurometabolite	MRS‐NAA, MRS‐Cr	53dps/48dpt (A)	*P* < 0.05^∗^
		Microglia activity	Iba+		*P* < 0.05^#^
		Angiogenesis	Ang1		
		Neurogenesis	Dcx+		

Tang et al. 2014 [23]	Sprague–Dawley rats	Infarct volume	CV staining	14dps/13dpt (A)	*P* < 0.05^∗^
		Apoptosis	TUNEL+/NeuN+		
		Angiogenesis	CD31+/BrdU+		*P* < 0.05^#^
		Neurogenesis	Dcx+/BrdU+		

Ma et al. 2015 [29]	C57BL/6 mice	Infarct volume	CV staining	9dps/7dpt (A)	*P* < 0.05^∗^

Yamashita et al. 2017 [32]	C57BL/6N mice	Astrogliosis	GFAP+	8dps/7dpt (A)	*P* < 0.05^∗^

Ziaee et al. 2017 [42]	Sprague	Infarct volume	H&E	31dps/28dpt (A)	*P* < 0.05^∗^
	Dawley rats	Apoptosis	Caspases activity TUNEL+		

Yu et al. 2018 [41]	Sprague	Astrogliosis	GFAP+	3dps/3dpt (hA)	*P* < 0.05^∗^
	Dawley rats	Microglia activity	Iba1		
		Autophagy	Beclin‐1, MAPILC3A		*P* < 0.05^#^
		Synaptogenesis	MAP‐2, GAP‐43, Synaptophysin		

Yao et al. 2015 [39]	Sprague	Neurometabolism	MRS–(NAA‐Cho)/Cr	14dps/12dpt (A)	*P* < 0.05^∗^
	Dawley rats				

Eckert et al. 2015 [33]	C57BL/6J	BBB stability	MMP‐9, ZO‐1, IgG staining	2dps/1dpt (A)	*P* < 0.001^∗^, *P* < 0.05^#^, *P* < 0.05^∗^
		Microglia activity	Iba+, mRNA‐[MCP‐1, MIP‐1*α*]		*P* < 0.001^∗^, *P* < 0.01^∗^,*P* < 0.001^∗^

Kondori et al. 2020 [36]	Wistar rats	Infarct volume	TTC	8dps/7dpt (A)	*P* < 0.01^∗^

Yin et al. 2023 [40]	C57BL6/j mice	Brain atrophy	CV	14dps/14dpt (hA)	*P* < 0.05^∗^
		Infarct volume		2dps/2dpt (hA)	
		Brain edema			
		BBB stability	IgG staining		
		Leukocytes	CD45+		*P* < 0.01^∗^
		Neut/mono	Ly6G+/Ly6C+		*P* < 0.05^∗^,*P* < 0.01^∗^
		Lymphoid cells	CD3*ε*+/NK+		*P* < 0.01^∗^,*P* < 0.001

Vonderwalde et al. 2020 [38]	SCID/beige mice	Synaptogenesis	Synaptophysin	32dps/28dpt (A)	*P* = 0.003^#^

Hermanto et al. 2018 [34]	Wistar rat	Microglia activity	Iba1+	32dps/30dpt (A)	*P* = 0.0211^∗^

Tahta et al. 2018 [47]	Sprague–Dawley rats	Infarct volume	MRI‐T2W	28dps/27dpt (A)	*P* < 0.0001^∗^
		Microglia activity	CD11+		
		Astrocytes	GFAP+		*P* < 0.0001^#^
		Inflammatory Response	CD5+		*P* = 0.038^#^

Lu et al. 2017 [45]	ICR mice	Infarct volume	CV	14dps/10dpt (A)	*P* < 0.01^∗^
		Angiogenesis	CD31+		*P* < 0.001^#^

*Note:* The phase of stroke was defined based on “the duration of stroke prior to treatment (N[dps]–N[dpt]): hyperacute (hA) = 0–24 h, acute (A) = 1–7 days, and subacute (sA) = 1 week–6 months, For infarct volume analysis, “^∗^” represents a significant decrease in infarct volume in the treatment group compared to the vehicle group.

Abbreviations; Ang 1, angiopoietin‐1; B4, isolectin B4; BDA, biotinylated dextran amine; BrdU, 5‐bromo‐2′‐deoxyuridine; CD, cluster of differentiation; Cho, choline; CID, severe combined immunodeficiency; Cr, creatine; CV, cresyl violet staining; DAB, diaminobenzidine staining; Dcx, doublecortin; dps, day(s) poststroke; dpt, day(s) posttreatment; EBE, Evans blue extravasation; ED, ectodysplasin; GAP‐43, growth‐associated protein 43; GFAP, glial fibrillary acidic protein; H&E, hematoxylin and eosin; Iba, ionized calcium‐binding adapter molecule; IgG, immunoglobulin G; Ly6C: lymphocyte Antigen 6 family member C; Ly6G: lymphocyte Antigen 6 family member G; MAP‐2, microtubule‐associated protein 2; MAPILC3A: microtubule‐associated protein 1 A/1B light chain 3A; MBP, myelin basic protein; MCP, monocyte chemotactic protein, MIP, macrophage inflammatory protein, MMP, matrix metalloproteinase; MRI, magnetic resonance imaging, MRS, magnetic resonance spectroscopy, NAA, N‐acetyl aspartate; NeuN, neuronal nuclei antigen; NG2, nerve/glial‐antigen 2; NK, natural killer; RECA‐1, rat endothelial cell antigen‐1; T2W, T2‐weighted; TTC, triphenyl tetrazolium chloride staining; TUNEL, TdT‐dUTP nick end labeling; SICR, Institute of Cancer Research; vWF, von Willebrand factor; ZO‐1, zonula Occludens‐1.

^#^A significant increase in the marker led to improvement in the measured structural outcome in the treatment group compared to the vehicle group.

^∗^A significant decrease in the outcome marker led to improvement in the measured structural outcome in the treatment group compared to the vehicle group.

### 3.4. Evidence of Advantageous Alteration in the Levels of Some Paracrine Factors

Some studies showed significant and advantageous alterations in the levels of paracrine factors (such as increased levels of neurotrophins, growth factors, and anti‐inflammatory cytokines, and decreased levels of proinflammatory cytokines and cell adhesion molecules) when NSPCs were transplanted during the acute phase of ischemic stroke. When treatment was delivered during the subacute phase of stroke, a similar outcome was observed [26]. Compared to vehicle treatment, NSPC transplantation increased the levels of neurotrophins (> BDNF, *p* < 0.001) [27, 33], but decreased the levels of proinflammatory cytokines (< TNF‐α and < IL‐1β, *p* < 0.001) [27] and cell adhesion molecules (< ICAM and < VCAM, *p* < 0.001) [27, 33] (Table 5).

**TABLE 5:** Evidence of advantageous alteration in the levels of some paracrine factors.

Study (year)	Immunobiochemical substance	Marker	Time point	*p* value
Doeppner et al. 2014 (19)	Neurotrophic factor	BDNF, GDNF	84dps/56dpt (sA)	*P* < 0.05^#^
	Growth factor	VEGF		

Ryu et al. 2016 [30]	Nuclear antigen	PCNA	14dps/13dpt (A)	*P* < 0.05^#^

Huang et al. 2014 (20)	Proinflammatory cytokines	TNF‐α, IL‐1β, IL‐6	2dps/1dpt (A)	2*p* < 0.001^∗^, *P* < 0.01^∗^
	Cell adhesion molecules	ICAM‐1, VCAM‐1		*P* < 0.001^∗^, *P* < 0.01^∗^
	Neurotrophic factor	BDNF		*P* < 0.001^#^

Baker et al. 2017 (17)	Neurotrophic factors	BDNF, GDNF	53dps/48dpt (A)	*P* < 0.05^#^
	Growth factors	NOG, NTF3		
	Anti‐inflammatory cytokines	IL‐10		
	Neurite branching mediator	RTN3		
	Proinflammatory cytokines	IL‐1β, IL‐6, CSF2		*P* < 0.05^∗^

Tang et al. 2014 [23]	Growth factors	VEGF	14dps/13dpt (A)	*P* < 0.05^#^

Yu et al. 2018 (34)	Proinflammatory cytokines	TNF‐α, leptin, L‐selectin,	3dps/3dpt (hA)	2*p* < 0.05^∗^, *P* < 0.01^∗^, *P* < 0.001^∗^
	Anti‐inflammatory cytokines	MCP‐1		
		TIMP‐1		*P* < 0.05^#^

Eckert et al. 2015 (26)	Proinflammatory cytokines	TNF‐α, IL‐1β	2dps/1dpt (A)	*P* < 0.01^∗^, *P* < 0.05^∗^
	Cell adhesion molecules	ICAM‐1, VCAM‐1		*P* < 0.01^∗^, *P* < 0.001^∗^
	Neurotrophic factor	BDNF		*P* < 0.001^#^

*Note:* The phase of stroke was defined based on “the duration of stroke prior to treatment” (N[dps]–N[dpt]): hyperacute (hA) = 0–24 h, acute (A) = 1–7 days, and subacute (sA) = 1 week–6 months.

Abbreviations: BDNF, brain‐derived neurotrophic factor; CSF2, colony‐stimulating Factor‐2; dps, day(s) poststroke; dpt, day(s) posttreatment; GDNF, glial cell line–derived neurotrophic factor; ICAM‐1, intercellular adhesion Molecule‐1; IL, interleukin; MCP‐1, monocyte chemoattractant Factor‐1; NOG, Noggin; NTF3, Neurotrophin‐3; PCNA, proliferating cell nuclear antigen; RTN3, Reticulon‐3; TIMP‐1, tissue inhibitor of Metalloproteinase‐1; TNF, tumor necrosis factor; VCAM‐1, vascular cell adhesion Molecule‐1; VEGF, vascular endothelial growth factor.

^#^means a significant increase in the marker led to increased levels of the immuno‐biochemical substance in the treatment group compared to the vehicle group.

^∗^means a significant decrease in the marker led to decreased levels of the immuno‐biochemical substance in the treatment group compared to the vehicle group.

## 4. Discussion

**FIGURE 2:**
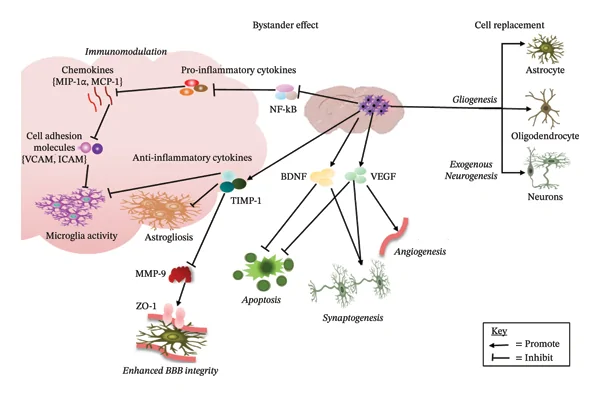
Schematic diagram illustrating the multimechanistic reparative pathways of NSPC therapy for ischemic stroke. NSPCs repair ischemic brain injury either by replacing neurons and neuroglial populations or by the pleiotropic bystander effect. Abbreviations: BDNF: brain‐derived neurotrophic factor, VEGF: vascular endothelial growth factor, MCP‐1: monocyte chemoattractant factor‐1, MIP: macrophage inflammatory protein‐1α, ICAM: intercellular adhesion molecule, VCAM: vascular cell adhesion molecule, TIMP‐1: tissue inhibitor of metalloproteinase‐1, MMP: matrix metalloproteinase, ZO‐1: zonula occludens‐1. NF‐κB: nuclear factor‐κB.

Amidst the burgeoning popularity of cellular therapy as a treatment modality for ischemic stroke, there is evidence suggesting that NSPCs could be a promising candidate for treating ischemic stroke. Moreover, NSPCs are generally regarded as safe, as they lack tumorigenic tendencies [49] and have demonstrated preclinical efficacy in restoring motor, sensory, and cognitive functions in animal models [50]. However, despite encouraging functional outcomes, the precise reparative mechanisms remain incompletely defined, and this limits the rational optimization of therapeutic strategies. Based on the evidence synthesized in this review, NSPC transplantation improved neurobehavioral outcomes through two overarching mechanisms, namely, (1) neuronal differentiation and replacement of lost cells, and (2) paracrine bystander effects mediated by secreted trophic, angiogenic, and immunomodulatory factors (Figure 2). Importantly, mounting preclinical evidence suggests that the latter mechanism is the consistent driver of recovery, whereas direct neuronal replacement occurs at a lower frequency. This distinction is crucial for guiding future therapeutic development, as it shifts the paradigm from a neuron‐centric to a paracrine‐centric one. These mechanisms are subsequently discussed.

### 4.1. Cell Replacement

Research has shown that the neurorestorative outcomes after NSPC transplantation were initially attributed primarily to the neuronal replacement role of the graft, based on early experimental evidence suggesting integration of grafted cells into host circuits [51]. Mostly, this was thought to result from NSPC‐mediated endogenous neurogenesis. In this review, some studies demonstrated that, compared to the vehicle, NSPC transplantation increased the number of BrdU + proliferating cells differentiating into multiple lineages, including mature neurons [26], immature neurons [26, 30], oligodendrocytes [28], and astroglial cells [30]. These findings confirm that NSPCs can contribute to both neuronal and glial cell pools. However, the magnitude of neuronal replacement appears modest across studies. While new neurons were detectable, the majority of transplanted or newly generated cells preferentially adopted glial rather than neuronal fates [26, 52]. This aligns with reports that functional recovery often occurred even when neuronal differentiation was sparse, suggesting that replacement alone is insufficient to explain behavioral improvements. Thus, the neurorestorative responses observed after transplantation likely involve a dual contribution, including (1) limited neuronal and glial replacement that aids remyelination and neurovascular stability, and (2) secretion of neurotrophic factors that stimulate endogenous neurogenesis and support synaptic plasticity [50, 53]. Importantly, this indicates that while cell replacement contributes to repair, it is neither the sole nor the dominant mechanism and must be considered within the broader context of NSPC paracrine activity.

### 4.2. Bystander Effects

According to Hao et al. (2014), enhancement of functional recovery ensues even in the absence of neurogenesis [54]. This finding, together with evidence from most studies included in this review, underscores that NSPC‐mediated repair is not exclusively dependent on neuronal differentiation but is instead strongly driven by a multifaceted paracrine bystander effect. This reparative mechanism involves dynamic crosstalk between grafted cells, host immune populations, and a wide repertoire of secretory factors such as cytokines, chemokines, growth factors, and neurotrophic factors. The pleiotropic reparative mechanisms conferred by the bystander effects of NSPCs are discussed below.

#### 4.2.1. Ameliorating Neuronal Death and Enhancing Synaptogenesis

NSPCs secrete neurotrophic factors such as brain‐derived neurotrophic factor (BDNF) that avert apoptosis of neurons by activating the PI3K/Akt pathway and the MAPK/ERK pathway [55, 56]. The downstream effect of these BDNF‐activated pathways, specifically the BDNF‐TrkB‐ERK pathway, enhances antiapoptotic BCL‐2 expression, while suppressing caspase‐3 activity [57]. The activation of the pathway ultimately stymies apoptosis of neuronal cells, which in turn salvages the neuronal population and culminates in a reduction of infarct volume. Consistent with this mechanism, several studies in our review reported reduced caspase activity and TUNEL + cells (indicators of apoptosis) and a concomitant reduction of infarct volume following NSPC transplantation. Beyond survival, BDNF mediates synaptogenesis and plays a cardinal role in cognitive plasticity [58]. A study demonstrated that BDNF knockout led to a substantial reduction of synaptophysin (a marker of synaptogenesis) [59], while overexpression of BDNF increased the level of synaptophysin [60]. These findings suggest that NSPCs improve recovery not only by reducing neuronal death but also by strengthening synaptic architecture. However, BDNF effects are dose‐ and context‐dependent, as excess BDNF or overexpression in certain CNS models has been linked to hyperexcitability, accelerated seizure activity, and neuronal damage [61–63].

#### 4.2.2. Enhancing Vascular Integrity

After ischemia, survival of salvaged neuronal tissue depends heavily on maintaining vascular patency and integrity in peri‐infarct regions. Transplanted NSPCs secrete vascular endothelial growth factor (VEGF) and angiopoietin‐1 (ang‐1), which are potent growth factors and mediators of angiogenesis. VEGF and ang‐1 act to increase vascular density and enhance vascular integrity, which keeps the brain well‐perfused while healing ensues. Tang et al. (2014) demonstrated that NSC transplantation in aged rats increased VEGF expression, enhanced microvessel density, and improved neurological recovery, confirming the centrality of angiogenesis to functional restoration. Doeppner et al. (2014) further showed that transplantation timing modulates vascular outcomes; acute delivery stabilized the BBB, whereas postacute delivery enhanced angiogenesis and long‐term remodeling. Additionally, to some extent, VEGF is antiapoptotic and mitogenic, capable of eliciting neurogenesis [64]. Yet, VEGF’s role is context‐dependent; while moderate levels promote repair, uncontrolled expression early after stroke may exacerbate edema and hemorrhage [65]. Thus, NSPC‐mediated angiogenesis appears beneficial but requires careful spatiotemporal regulation. Collectively, these findings suggest that NSPC‐induced vascular repair is a cornerstone of the bystander effect, though its therapeutic optimization may require adjunct strategies such as controlled VEGF delivery or combination with endothelial progenitor cells.

#### 4.2.3. Immunomodulation

Immunomodulation is also a hallmark of enhanced poststroke recovery [66]. Inflammatory responses are evident during the acute phase of ischemic stroke, and microglia are known to mediate these responses [67]. Microglia release both proinflammatory (such as IL‐1β and TNF‐α) and anti‐inflammatory cytokines. When cytokine release is tilted toward the former, inflammation could progress to a chronic state. Proinflammatory cytokines activate nuclear factor‐κB (NF‐κB), an inducible transcription factor in the brain, which in turn leads to the downstream production of chemokines (MIP‐1, MCP‐1*α*) (Figure 2). The chemokines act in concert with different adhesion molecules (such as ICAM and VCAM) to recruit immune cells to the infarct site. These immune cells clear cellular debris and toxins at the expense of causing collateral damage. The transplanted NSPCs counteract this by attenuating NF‐κB activation [68], which reduces downstream chemokine production and limits excessive immune cell recruitment to the infarct core. Additionally, NSPCs secrete cytokines and exosomes that promote a shift in microglial phenotype from proinflammatory M1 to anti‐inflammatory M2 states [69]. Similar effects have been observed in astrocytes, with NSPCs promoting an A2 (neuroprotective) rather than an A1 (neurotoxic) profile [70]. Not all studies, however, observed sustained immunomodulatory benefits. While most of the included articles in this review reported reductions in proinflammatory cytokines and microglial activity during the acute and subacute phases, other studies outside our pool have demonstrated that these effects may be transient. Evidence from delayed transplantation and chronic stroke models indicates that reductions in inflammatory markers often decline at later stages [71–74]. Future studies should, therefore, assess whether repeated or combined interventions are necessary to maintain long‐term benefits. Nevertheless, the ability of NSPCs to recalibrate immune responses remains one of the strongest and most consistent contributors to functional recovery.

#### 4.2.4. Reducing Astrocytic Scar (Astrogliosis) Formation

Astrocytes are resident‐supporting cells in the brain and are involved in the pathogenesis of ischemic stroke [75]. During the subacute phase of ischemic stroke, astrocytes contribute to astrocytic scar formation (astrogliosis), which is evidenced at least by an increase in GFAP expression; astrogliosis significantly impairs neurorestoration [76]. Also, during ischemic stroke, astrocytes are responsible for ablating the excitatory neurotransmitter (glutamate) that triggers excitotoxicity [77]. This review found that NSPC transplantation decreased the number of GFAP + astrocytes, consistent with diminished astrogliosis and improved tissue permissiveness for regeneration. In contrast, Tahta et al. (2018) reported an increase in GFAP + astrocytes posttransplantation, attributed to NSPCs differentiating into astrocytic lineages [47]. These reports suggest that NSPCs may elicit neurorestorative responses either by differentiating into neuroprotective GFAP + astrocytes [23, 47] or reducing astrogliosis (decreased GFAP + astrocytes) [41]. Indeed, recent literature distinguishes between neurotoxic A1 astrocytes (C3+/GFAP+) and neuroprotective A2 astrocytes (S100A10+/GFAP+), showing that ischemic injury induces both phenotypes but with differing functional outcomes [78–81]. Without such subtype markers, reliance on GFAP expression alone may obscure whether astrocytic responses are beneficial or maladaptive. This heterogeneity indicates that NSPC effects on astrogliosis are context‐dependent. Future work should employ refined phenotyping to determine whether NSPCs preferentially drive neuroprotective astrocyte responses or suppress neurotoxic ones. The therapeutic implication is that modulating astrocytic phenotype, rather than simply reducing astrocyte numbers, may be a more effective strategy for harnessing NSPC benefits.

#### 4.2.5. Improving Blood–Brain Barrier Integrity

Ischemic stroke is characterized by increased permeability of the BBB, leading to diapedesis, hemorrhage, and vasogenic edema [82]. This increased BBB permeability is mediated by the activity of matrix Metalloproteinase‐9 (MMP‐9) [27, 33]. Studies have shown that MMP‐9 destroys the tight junction protein zonula occludens‐1 (ZO‐1) to destabilize the BBB [27]. NSPC transplantation has been shown to enhance BBB integrity, in part by upregulating tissue inhibitor of metalloproteinase‐1 (TIMP‐1), a natural inhibitor of MMP‐9 [83] (Figure 2). By stabilizing tight junctions, transplanted NSPCs reduce paracellular leakage, limit immune cell extravasation, and prevent secondary injury. Doeppner et al. (2014) further demonstrated that NSPC delivery during the acute phase improved BBB stability, supporting the idea that timing is critical; early intervention maximizes neuroprotection, whereas delayed delivery favors remodeling. Although robust across preclinical models, BBB repair remains underexplored in chronic stroke contexts, and it is unclear whether such effects persist long‐term. Translationally, enhancing BBB stabilization may be an important adjunct mechanism explaining functional gains after NSPC therapy, especially in patients at risk of hemorrhagic transformation.

### 4.3. Translational Considerations and Clinical Challenges

Despite consistent preclinical efficacy, NSPC‐based therapies have shown more modest and variable benefit when tested in stroke patients, echoing the broader history of translational failure from preclinical stroke models to clinical efficacy [84, 85]. Several factors identified in this review may help explain this gap. First, immunogenicity differed substantially across included studies: 14 (56%) used xenogeneic, and 11 (44%) used allogeneic NSPC sources, yet the functional consequences of this distinction were rarely analyzed within individual studies. Xenogeneic neural grafts elicit both innate and adaptive (T‐cell–mediated) immune responses in the host brain, whereas allogeneic grafts predominantly trigger innate immunity alone [86]; this distinction carries direct implications for graft survival, the durability of paracrine signaling, and the immunosuppressive regimens required for clinical translation, yet it was seldom controlled for explicitly in the preclinical literature reviewed here. Second, delivery routes varied widely, from intracerebral (48%) to intravenous, intrahippocampal, intraventricular, epidural, and intra‐arterial administration. Intracerebral/stereotactic delivery maximizes local cell retention but is invasive and not readily scalable to the acute clinical stroke pathway, whereas systemic (intravenous) delivery is more clinically feasible but delivers a smaller proportion of transplanted cells to the injury site. Third, early‐phase human trials of NSPC‐related therapies have themselves shown safety with only modest and inconsistent efficacy: the first‐in‐man PISCES trial of intracerebrally delivered allogeneic human NSCs (CTX0E03) in chronic stroke patients demonstrated safety and preliminary signals of neurological improvement without immunosuppression [87], whereas a recent phase II trial of intravenous allogeneic umbilical cord blood infusion for ischemic stroke found no significant difference in functional outcome between treatment and placebo groups [88]. These observations underscore that the paracrine and immunomodulatory mechanisms identified in this review, while robust in controlled preclinical settings, may be attenuated in the more heterogeneous, comorbid, and immunologically intact human stroke population, and that immunogenicity and delivery route should be treated as primary translational variables in future preclinical work rather than incidental study characteristics [89].

### 4.4. Limitations

Several limitations should be considered when interpreting the findings of this review. First, only 25 studies met the stringent eligibility criteria, which may have limited the breadth of evidence available for synthesis. Second, considerable heterogeneity existed among studies with respect to animal species, stroke models, NSPC sources, transplantation protocols, outcome measures, and follow‐up periods, precluding quantitative meta‐analysis. Consequently, the conclusion that paracrine and immunomodulatory mechanisms predominate over direct neuronal replacement rests on the breadth and consistency of narrative evidence rather than a formal, effect‐size‐weighted quantitative comparison, and should be interpreted accordingly: direct neurogenesis was explicitly quantified in only 5 of the 25 included studies, whereas angiogenic, immunomodulatory, and biochemical/paracrine outcomes were reported across the substantial majority of the remaining studies. Third, most included studies used young male animals, limiting the generalizability of the findings to females, older populations, and clinically relevant comorbidity profiles. Fourth, publication bias cannot be excluded because studies reporting negative or neutral outcomes are less likely to be published. Finally, all included studies were preclinical in nature; therefore, caution should be exercised when extrapolating these findings to human stroke patients.

### 4.5. Recommendations

Building on the evidence synthesized in this review, future research should focus on strategies that enhance and harness paracrine mechanisms, including preconditioning NSPCs to upregulate trophic factor release or combining transplantation with the exogenous administration of key mediators such as BDNF and VEGF. Incorporating sex as a biological variable is essential, given the modulatory effects of estrogen on neuroprotection. Likewise, the inclusion of comorbidities such as hypertension and diabetes in preclinical models would better reflect the clinical stroke population and improve translational relevance. Finally, novel approaches such as biomaterial scaffolds, gene‐edited NSPCs resistant to hostile microenvironments, and delayed transplantation paradigms should be explored to address practical limitations and extend therapeutic windows. Advancing research in these directions will accelerate the translation of NSPC‐based therapies into safe and effective interventions for human ischemic stroke.

## 5. Conclusion

This review highlights promising biological processes for future mechanistic investigation and supports the development of targeted strategies to enhance the therapeutic efficacy of NSPC‐based interventions. NSPC transplantation improves neurological outcomes in animal models of ischemic stroke consistently through paracrine bystander effects that mitigate apoptosis, enhance synaptogenesis, promote angiogenesis, stabilize the BBB, and modulate inflammatory responses. Although NSPCs can differentiate into neurons and glia, the frequency of neuronal replacement is limited, and differentiation is often skewed toward astrocytic fates. Conflicting reports on astrocytic outcomes underscore the need for better phenotypic characterization to distinguish between neuroprotective and neurotoxic subtypes. Nevertheless, the modest and inconsistent efficacy observed in early‐phase human trials indicates that immunogenicity, delivery route, and other translational variables identified in this review must be resolved before these paracrine‐centric mechanisms can be reliably harnessed in clinical practice. Collectively, these findings suggest that while neuronal replacement contributes modestly, it is the pleiotropic paracrine actions of NSPCs that play a central role in their reparative efficacy.

## Author Contributions

Joel Karikari Nyarkoh: conception, study design, and figure design. Joel Karikari Nyarkoh and Moses Tetteh Larnyoh: collection and/or assembly of data. Samuel Badu Nyarko: resolving divergence between data extractors. Joel Karikari Nyarkoh, Ernest Amponsah Asiamah, Gabriel Kotam Pezahso, Obed Akorli Mawunyo, Moses Tetteh Larnyoh, and Bernard Nyarko: manuscript writing. Joseph Boachie, Ernest Amponsah Asiamah, Samuel Badu Nyarko, Christian Dzuvor, Joel Karikari Nyarkoh, and Moses Tetteh Larnyoh: revision of the manuscript for important intellectual content.

## Funding

The current study did not receive financial support from funding bodies or sponsors.

## Disclosure

All authors read and approved the final manuscript.

## Ethics Statement

The authors have nothing to report.

## Consent

The authors have nothing to report.

## Conflicts of Interest

The authors declare no conflicts of interest.

## Data Availability

The data that support the findings of this study are available from the authors upon reasonable request.
